# Stereotactic body radiotherapy for oligoprogression with or without switch of systemic therapy

**DOI:** 10.1016/j.ctro.2024.100748

**Published:** 2024-02-23

**Authors:** Jonas Willmann, Eugenia Vlaskou Badra, Selma Adilovic, Maiwand Ahmadsei, Sebastian M. Christ, Stephanie Tanadini-Lang, Michael Mayinger, Matthias Guckenberger, Nicolaus Andratschke

**Affiliations:** Department of Radiation Oncology, University Hospital Zurich, University of Zurich, Rämistrasse 100, 8091 Zurich, Switzerland

## Abstract

**Background:**

Oligoprogression is defined as cancer progression of a limited number of metastases under active systemic therapy. The role of metastasis-directed therapy, using stereotactic body radiotherapy (SBRT), is controversial as is the continuation versus switch of systemic therapy. We report outcomes of oligoprogressive patients after SBRT, and compare those patients that continued or switched their current line of systemic therapy.

**Material/Methods.:**

We included patients who developed up to 5 progressive extracranial metastases under systemic therapy for any solid organ malignancy and were treated with SBRT to all lesions at our institution between 01/2014 and 12/2019. Overall survival (OS) and progression-free survival (PFS) were analyzed using the Kaplan-Meier method, and the interval to the next systemic therapy line determined using cumulative incidence functions. Multivariable Cox regression models were used to analyze the influence of baseline and post-progression variables on OS, PFS and survival with the next systemic therapy after SBRT.

**Results:**

Among 135 patients with oligoprogressive disease of which the most common primary tumor was lung cancer (n = 46, 34.1 %), 96 continued their current line of systemic therapy after oligoprogression. Among 39 who switched systemic therapy, 28 (71.8 %) paused or discontinued, while 11 (28.2 %) immediately started another systemic treatment. After a median follow-up of 27.2 months, patients that switched and those who continued systemic therapy after oligoprogression had comparable median OS (32.1 vs. 38.2 months, p = 0.47) and PFS (4.3 vs. 3.4 months, p = 0.6). The intervals to the next systemic therapy line were comparable between both cohorts (p = 0.6). An ECOG performance status of 2 and immediately starting a new systemic therapy after oligoprogression were associated with a poorer survival without next systemic therapy, while the de-novo OMD state was associated with better survival without next systemic therapy compared to the induced state.

**Conclusion:**

Oncological outcomes of patients that continued or switched systemic therapy after SBRT for oligoprogression were comparable, potentially indicating that further lines of treatment may be safely delayed in selected cases.

## Highlights

• We analyzed oligoprogressive patients treated with SBRT and continued or switched systemic therapy.
• Patients on targeted or immunotherapy more frequently continued systemic therapy.
• Patients that continued or switched on their systemic therapy had comparable OS and PFS.
• The interval to the next systemic therapy line was comparable in both patient groups.

## Introduction

Oligometastatic disease (OMD) represents a distinct intermediate state in the cancer disease continuum, characterized by a limited number of metastatic lesions which can be targeted with local therapies [1]. This is in contrast to polymetastatic disease, where a higher burden of metastatic disease exists, often spread across multiple organs.

Oligoprogressive disease, characterized by limited progression in an otherwise controlled systemic disease, poses a unique challenge in the realm of OMD [2]. This clinical scenario, where a limited number of metastatic sites progress despite an otherwise effective systemic therapy regimen, warrants a balance between local ablative and systemic treatment strategies. Patients with oligoprogressive disease may present with intrinsic (primary) or acquired (secondary) resistance to certain systemic treatments. The underlying molecular resistance mechanisms have distinct implications for the systemic and local treatment strategy. Intrinsic resistance results in an immediate inefficacy of a therapeutic agent. Acquired resistance, i.e. progression after an initial clinical benefit, may occur through various mechanisms, depending on the target of the employed systemic therapy, due to the evolutionary clonal selection of resistant tumor cells [3], [4], [5], [6], [7].

The advent of stereotactic body radiotherapy (SBRT), with its ability to deliver highly conformal, ablative doses of radiation to tumor targets with an acceptable safety profile [8], has revolutionized the local treatment of oligoprogression [9]. However, the optimal combination of metastasis-directed SBRT and systemic therapy for patients with oligoprogression remains a subject of ongoing debate - particularly, when to switch systemic therapy [10].

To contribute to the growing body of evidence defining the role of SBRT in combination with systemic therapy in patients with oligoprogression, we compared oncological outcomes in patients who switched their systemic therapy following SBRT for oligoprogression with those who continued on their current systemic therapy. An additional objective was to analyze the patterns of subsequent systemic therapy.

## Material/Methods

### Study design and patient selection

This retrospective single-center study assessed consecutive oligometastatic patients treated with metastasis-directed SBRT between January 2014 and December 2019 at the University Hospital Zurich. Inclusion criteria were oligoprogression, i.e., the development of new or progressive oligometastases under active systemic therapy, with a maximum of 5 extracranial metastases, all treated with SBRT. There were no restrictions regarding primary tumor entities. OMD states were determined according to the ESTRO and European Organisation for Research and Treatment of Cancer (EORTC) classification of OMD [2]. In brief, all patients had oligoprogressive disease, defined as development of OMD under active systemic therapy. Patients were further classified as having metachronous oligoprogression, i.e., the first diagnosis of OMD (more than 6 months after initial diagnosis of non-metastatic cancer), and repeat oligoprogression, after a previous history of OMD, and induced oligoprogression, after a history of polymetastatic disease.

Two subgroups of patients were defined: those that continue their current systemic therapy beyond oligoprogression for at least one month, and those that switched systemic therapy within one month of oligoprogression. The latter include patients that immediately start the next line of systemic therapy, and those that discontinue or pause systemic therapy, and may later on start a next line.

This study followed the STROBE guideline for reporting of cohort studies and was approved by the institutional ethics board as well as the state ethics committee (BASEC ID 2018–01794).

### Treatment and follow-up

For the purpose of this study, SBRT was defined as the application of conformal treatment planning, image-guidance and stereotactic patient setup, using hypofractionated treatment application and inhomogeneous dose prescription. Non-ablative, palliative treatment regimens, e.g. 8 Gy in a single fraction or 5 x 4 Gy (homogeneously prescribed), were excluded. Patients were followed up after SBRT with regular imaging and clinical assessment according to institutional guidelines.

Generally, OMD patients are followed up every three months for the first year after radiotherapy and every six months thereafter or until progression, with clinical assessment and the imaging modality deemed appropriate by the treating primary oncologist and depending on the primary tumor location and histology, preferably FDG-PET/CT scan. Patients with oligometastatic prostate cancer receive Prostate-Specific Antigen (PSA) tests every three months and PSMA-PET/CT or /MRI scans in case of biochemical recurrence.

The decision to continue the current systemic therapy beyond oligoprogression that was treated with SBRT was generally made in a multidisciplinary tumor board. In the absence of prospective randomized data, no specific criteria were defined to select patients for continuing systemic therapy beyond oligoprogression. Generally, the patients’ performance status, disease burden and progression dynamics, side effects of the current systemic therapy and expected toxicity profile and efficacy of the next systemic therapy line, as well as its potential impact on the patients’ quality of life and the patients’ preference were considered.

### Statistical analysis

For descriptive statistics, median and interquartile range (IQR) were used to describe continuous patient and treatment data variables and absolute counts and percentages for categorical data. Group comparisons were performed with the use of Fisher’s exact test or chi-square tests for categorical variables and Mann-Whitney U tests for continuous variables, as appropriate. Overall survival (OS) and progression-free survival (PFS) were measured from the end of SBRT. Kaplan-Meier curves were used to present time-to-event outcomes, and pairwise log-rank tests were used to compare differences for statistical significance.

The interval from SBRT to the initiation of the next line of systemic therapy was analyzed by comparing the cumulative incidence for starting a new line of systemic therapy or death without starting a new systemic therapy, assuming competing risks. For determining the interval to and survival without the next line of systemic therapy after oligoprogression, the following criteria were applied: For patients *continuing* their previous systemic therapy post-oligoprogression, the subsequent line was counted. In cases where systemic therapy was *discontinued or paused* after oligoprogression, the next line was counted upon restarting systemic therapy. Lastly, for those *immediately switching* to a different systemic therapy following oligoprogression, the start of the following therapy marked the start of the next systemic treatment line.

Gray’s test was used to compare differences between patients that continue and switched their current systemic therapy after SBRT for statistical significance.

Multivariate Cox proportional hazards models were applied to assess the impact of different baseline (age, OMD state, primary tumor, systemic therapy after OMD diagnosis) and post-progression (repeat SBRT) variables on OS, PFS and survival without next systemic therapy.

The threshold for statistically significant difference was set at p ≤ 0.05. All statistical analyses were performed in R (R version 4.03.00; R Development Core Team), with the “survival”, “survminer”, “cmprsk”, “tidycmprsk”, “clinfun” and “finalfit” packages.

## Results

### Patient characteristics

Among 545 patients treated with SBRT for oligometastatic disease at our institution, 135 (24.8 %) presented with oligoprogression, i.e. developed their metastases under active systemic therapy, and were included in this study. We distinguished two subgroups based on the handling of systemic therapy: 96 patients (71.1 %) continued their current systemic therapy, while 39 patients (28.9 %) switched their systemic therapy, i.e. either immediately started a new treatment or discontinued/paused systemic therapy. The median age was 64.5 years (interquartile range [IQR], 55.8 to 71.4), and 53 patients (39.3 %) were female. Lung cancer was the most common primary (n = 46, 34.1 %), and numerically though not significantly more common in patients that continued their previous systemic therapy. The majority (n = 69, 51.1 %) had induced oligoprogression. Repeat oligoprogression occurred in 34 patients (25.2 %) and metachronous oligoprogression in 32 patients (23.7 %).

The distribution of the different states of oligometastatic disease differed in the two subgroups (p = 0.02): among patients that continued their current systemic therapy, 57.3 % (n = 55) presented with induced oligoprogression, while only 35.9 % (n = 14) of patients that switched systemic therapy had induced oligoprogression. On the other hand, 41.0 % (n = 16) of patients that switched systemic therapy had repeat oligoprogression, compared with 18.8 % (n = 18) of those that continued the current systemic therapy.

Fifty patients (37 %) in the entire cohort were treated with repeat SBRT to all lesions at distant progression. The rate of patients receiving repeat SBRT did not differ for those continuing and switching systemic therapy initially. Patient characteristics in the entire cohort and the subgroups that switched or continued their current systemic therapy are depicted in Table 1.

**Table 1:** Baseline patient characteristics for the entire group, and subgroups that switch or continue systemic therapy after SBRT. Data are in n (%) or median (IQR). P-values comparing subgroups that switch and continue systemic therapy.

		Entire cohort	Switch systemic therapy	Continue systemic therapy	*p-value*
	***Total N (%)***	*135 (100.0)*	*39 (28.9)*	*96 (71.1)*	
**Age (years)**	Median (IQR)	64.5 (55.8 to 71.4)	67.8 (58.7 to 72.1)	64.2 (55.0 to 69.9)	0.18
**Sex**	Male	82 (60.7)	23 (59.0)	59 (61.5)	0.94
	Female	53 (39.3)	16 (41.0)	37 (38.5)	
**Primary tumor**	Lung	46 (34.1)	8 (20.5)	38 (39.6)	0.10
	Colorectal	6 (4.4)	3 (7.7)	3 (3.1)	
	Breast	12 (8.9)	3 (7.7)	9 (9.4)	
	Melanoma	25 (18.5)	6 (15.4)	19 (19.8)	
	Prostate	6 (4.4)	1 (2.6)	5 (5.2)	
	Urogenital	9 (6.7)	2 (5.1)	7 (7.3)	
	Head and Neck	6 (4.4)	3 (7.7)	3 (3.1)	
	Gastrointestinal	11 (8.1)	5 (12.8)	6 (6.2)	
	Other	14 (10.4)	8 (20.5)	6 (6.2)	
**OMD state**	Induced oligoprogression	69 (51.1)	14 (35.9)	55 (57.3)	**0.02**
	Metachronous oligoprogression	32 (23.7)	9 (23.1)	23 (24.0)	
	Repeat oligoprogression	34 (25.2)	16 (41.0)	18 (18.8)	
**ECOG PS**	0	64 (47.4)	16 (41.0)	48 (50.0)	0.54
	1	23 (17.0)	8 (20.5)	15 (15.6)	
	2	6 (4.4)	3 (7.7)	3 (3.1)	
	Unknown	42 (31.1)	12 (30.8)	30 (31.2)	
**Primary tumor controlled**	Not controlled	11 (8.1)	4 (10.3)	7 (7.3)	0.82
	Controlled	124 (91.9)	35 (89.7)	89 (92.7)	
**Number of metastases**	1	89 (65.9)	26 (66.7)	63 (65.6)	0.57
	2	27 (20.0)	9 (23.1)	18 (18.8)	
	3	14 (10.4)	2 (5.1)	12 (12.5)	
	4	5 (3.7)	2 (5.1)	3 (3.1)	
**Cumulative metastases volumes**	Mean (SD)	15.0 (5.0 to 40.2)	18.4 (3.9 to 57.6)	14.5 (5.3 to 35.6)	**0.04**
**Involved organs**	Single	118 (87.4)	36 (92.3)	82 (85.4)	0.42
	Multiple	17 (12.6)	3 (7.7)	14 (14.6)	
**Staging imaging**	CT	30 (22.2)	13 (33.3)	17 (17.7)	0.14
	PET	101 (74.8)	25 (64.1)	76 (79.2)	
	MRI	4 (3.0)	1 (2.6)	3 (3.1)	
**Repeat SBRT**	Yes	50 (37.0)	13 (33.3)	37 (38.5)	0.71
	No	85 (63.0)	26 (66.7)	59 (61.5)	

Abbreviations: OMD: oligometastatic disease; ECOG PS: Eastern Cooperative Oncology Group performance status.

### Patterns of initial and subsequent systemic therapy

We compared systemic therapy before and after oligoprogression in the two previously defined subgroups. Patients that continued their current systemic therapy were more heavily pretreated (2 or more previous treatment lines in n = 72, 75.0 %) compared with patients that switched systemic therapy within a month of oligoprogression (2 or more previous treatment lines in n = 18, 46.2 %; p = 0.003). The type of systemic therapy administered at diagnosis of oligoprogression was also different between the two groups. A higher proportion of patients that continued their systemic therapy received targeted therapy (n = 36, 37.5 % vs. n = 7, 17.9 %; p = 0.045) and immunotherapy (n = 32, 33.3 % vs. n = 3, 7.7 %; p = 0.004).

Among patients that switched their systemic therapy within one month after oligoprogression, 28 (71.8 %) paused or discontinued, while 11 (28.2 %) immediately started another systemic treatment. Eventually, 64.1 % (n = 25) of the patients who changed systemic therapy started a subsequent systemic treatment line, while 15.4 % (n = 6) died without having commenced a subsequent line and 20.5 % (n = 8) were alive at last follow-up without a subsequent line. The adjustment of subsequent systemic treatments were comparable in patients that continued their current systemic therapy after oligoprogression, with 61.5 % (n = 59) starting a subsequent line and 11.5 % (n = 11) having died without starting a subsequent line. Remarkably, 27 % (n = 26) of patients who continued systemic therapy were alive and had not started the next systemic therapy.

In the subsequent treatment line, the types of agents administered were comparable among patients that switched or continued systemic therapy after oligoprogression. Details about systemic therapy before and after diagnosis of oligoprogression are depicted in Table 2.

**Table 2:** Systemic therapy characteristics before and after SBRT. Values are in n and %.

	**Switch systemic therapy**	**Continue systemic therapy**	**p-value**
Total N (%)	39 (28.9)	96 (71.1)	
**Number of systemic treatment lines**			**0.003**
2 or more	18 (46.2)	72 (75.0)	
1	21 (53.8)	24 (25.0)	
**Type of systemic therapy at OMD diagnosis**			
Targeted therapy	7 (17.9)	36 (37.5)	**0.045**
Immunotherapy	3 (7.7)	32 (33.3)	**0.004**
Chemotherapy	15 (38.5)	23 (24.0)	0.14
Endocrine therapy	2 (5.1)	11 (11.5)	0.42
**Adjustment of systemic therapy after oligoprogression**			<0.001
Continue current therapy	0 (0.0)	96 (100.0)	
Discontinue/pause therapy	28 (71.8)	0 (0.0)	
Start new therapy	11 (28.2)	0 (0.0)	
**Subsequent lines of systemic therapy**			0.655
Started subsequent line of systemic therapy	25 (64.1)	59 (61.5)	
Alive without subsequent line of systemic therapy	8 (20.5)	26 (27.1)	
Died without subsequent line of systemic therapy	6 (15.4)	11 (11.5)	
**Type of subsequent systemic therapy**			
Targeted therapy	11 (28.2)	21 (21.9)	0.58
Immunotherapy	9 (23.1)	12 (12.5)	0.20
Chemotherapy	10 (25.6)	26 (27.1)	1.00
Endocrine therapy	1 (2.6)	5 (5.2)	0.83

### Oncological outcomes

**Fig. 1:**
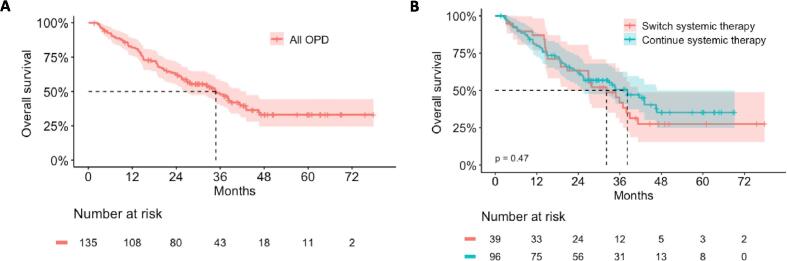
Overall survival in A) the entire cohort of patients with oligoprogressive disease (OPD) and B) comparing patients that continue their previous systemic therapy (blue) or switch systemic therapy (red) after SBRT. Band indicates 95% confidence interval.

After a median follow-up of 27.2 months, 76 patients died and 120 either progressed or died. In the entire cohort, the median OS was 34.8 months (95 %-confidence interval [CI] 27.0–43.0) (Fig. 1A). Patients that continued systemic therapy and patients that switched systemic therapy after SBRT had a comparable median OS of 38.2 months (95 %-CI 25.3–46.7) and 32.1 months (95 %-CI 20.8–41.2), respectively (p = 0.47) (Fig. 1B). The 1- and 2-year OS rates were 87.0 % (95 %-CI 76.9–98.3) and 63.2 % (95 %-CI 49.6–80.6) for patients that switched, and 79.9 % (95 %-CI 72.3–88.4) and 62.5 % (95 %-CI 53.4–73.2) for patients that continued systemic therapy. An exploratory analysis of OS in the three subgroups of patients that continued systemic therapy, started a new line, or discontinued/paused systemic therapy after oligoprogression is shown in the supplementary material, revealing no significant differences (Fig. A1).

**Fig. 2:**
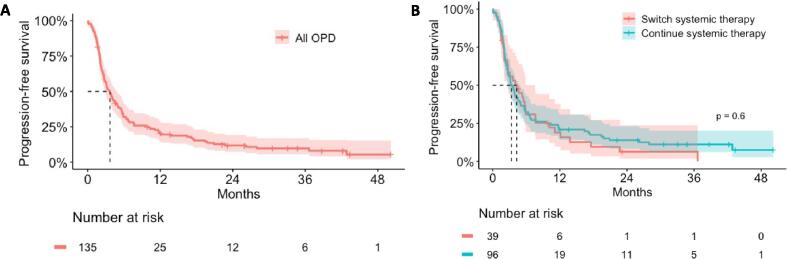
Progression-free survival in A) the entire cohort of patients with oligoprogressive disease (OPD) and B) comparing patients that continue their previous systemic therapy (blue) or switch systemic therapy (red) after SBRT. Band indicates 95% confidence interval.

The median PFS in the entire cohort was 3.7 months (95 %-CI 2.8–4.8) (Fig. 2A). There was no significant difference in median PFS of patients that switched (4.3 months, 95 %-CI 2.7–7.6) or continued systemic therapy (3.4 months, 95 %-CI 2.7–4.8; p = 0.6) (Fig. 2B). At 6 and 12 months, the PFS rates were 30.9 % (95 %-CI 19.0–50.1) and 18.9 % (95 %-CI 9.5–37.6) for patients that switched, and 32.3 % (95 %-CI 24.2–43.1) and 20.8 % (95 %-CI 14.1–30.8) for patients that continued systemic therapy.

**Fig. 3:**
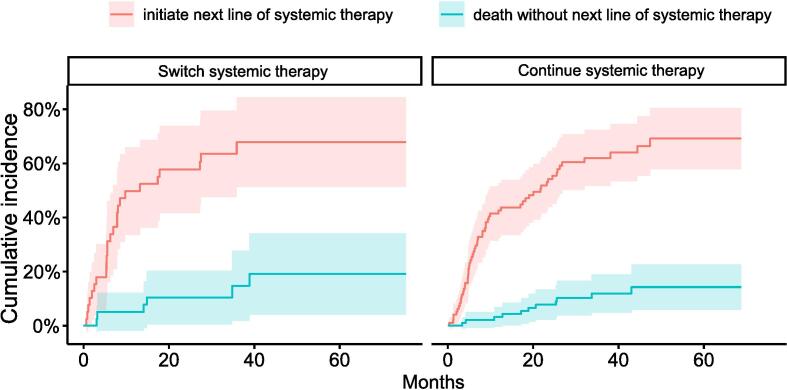
Cumulative incidence of initiating the next line of systemic therapy in oligoprogressive patients that continue or switch on systemic therapy after SBRT, accounting for the competing risk of death without changing systemic therapy.

The interval until the next line of systemic therapy was compared in patients who switched and continued their current systemic therapy after initial SBRT for oligoprogression. The 1- and 2-year cumulative incidence of initiating a new line of systemic therapy was 42.6 % (95 %-CI 32–52 %) and 54.2 % (95 %-CI 43–64 %), respectively, in patients that continued systemic therapy after SBRT, and was comparable (Gray’s test p = 0.6) to patients who switched systemic therapy after SBRT, with a cumulative incidence at 1 and 2 years of 49.8 % (95 %-CI 33–65 %) and 57.7 % (95 %-CI 40–72 %), respectively (Fig. 3).

### Multivariable analysis of factors influencing overall survival, progression-free survival, and survival without next systemic therapy

We investigated which baseline and post-progression characteristics influenced oncological outcomes after SBRT for oligoprogression (Table 3). Patients with an ECOG performance status of 2 showed a significantly increased hazard ratio (HR) of 2.91 (95 % CI: 1.09–7.81, p = 0.03) compared to patients with lower ECOG performance status, indicating a poorer survival without next systemic therapy. Both de-novo and repeat OMD states were associated with better survival without next systemic therapy compared to the induced state. The HR for de-novo was 0.52 (95 % CI: 0.30–0.89, p = 0.02) and for repeat was 0.58 (95 % CI: 0.33–1.01, p = 0.06), although the latter was above the threshold of statistical significance. Immediately starting a new systemic therapy after OMD diagnosis was significantly associated with worse survival without next systemic therapy, with an HR of 2.46 (95 % CI: 1.22–4.98, p = 0.01), compared with continuing systemic therapy. Other variables such as age, primary tumor type, and application of repeat SBRT did not demonstrate a statistically significant association with survival without next systemic therapy. The associating of these variables with OS and PFS, as well as survival without next systemic therapy, is shown in Table 3.

**Table 3:** Multivariable Cox regression models on the association of baseline and post-progression variables with overall survival, progression-free survival and the survival without next systemic therapy after SBRT. Abbreviations: OMD: oligometastatic disease; ECOG PS: Eastern Cooperative Oncology Group performance status; HR: hazard ratio; SD: standard deviation.

			HR (95 %-CI, p-value)		
Variable		n (%)	Overall survival	Progression-free survival	Survival without next systemic therapy
Age (years)	Mean (SD)	62.9 (11.9)	1.00 (0.98–1.03, p = 0.68)	1.00 (0.98–1.02, p = 0.98)	0.98 (0.96–1.00, p = 0.06)
ECOG PS	0	64 (47.4)	–	–	–
	1	23 (17.0)	1.33 (0.68–2.59, p = 0.41)	1.12 (0.64–1.97, p = 0.68)	1.44 (0.81–2.57, p = 0.22)
	2	6 (4.4)	**4.53 (1.42**–**14.41, p = 0.01)**	**5.36 (2.14**–**13.41, p < 0.001)**	**2.91 (1.09**–**7.81, p = 0.03)**
OMD state	Induced	69 (51.1)	–	–	–
	De-novo	32 (23.7)	**0.37 (0.19**–**0.72, p = 0.003)**	**0.44 (0.27**–**0.73, p = 0.001)**	**0.52 (0.30**–**0.89, p = 0.02)**
	Repeat	34 (25.2)	**0.36 (0.19**–**0.70, p = 0.003)**	**0.59 (0.36**–**0.99, p = 0.04)**	0.58 (0.33–1.01, p = 0.06)
Systemic therapy after oligoprogression	Continue current therapy	96 (71.1)	–	–	–
	Starting new therapy	11 (8.1)	1.73 (0.78–3.83, p = 0.18)	1.38 (0.70–2.73, p = 0.36)	**2.46 (1.22**–**4.98, p = 0.01)**
	Discontinue therapy	28 (20.7)	1.02 (0.54–1.94, p = 0.95)	0.95 (0.57–1.60, p = 0.85)	1.29 (0.73–2.30, p = 0.38)
Primary tumor	Lung	46 (34.1)	–	–	–
	Other	64 (47.4)	1.28 (0.74–2.20, p = 0.36)	1.16 (0.74–1.81, p = 0.51)	1.04 (0.64–1.69, p = 0.88)
	Melanoma	25 (18.5)	0.59 (0.29–1.23, p = 0.16)	0.99 (0.57–1.72, p = 0.98)	0.90 (0.50–1.64, p = 0.74)
Repeat SBRT	Yes	50 (37.0)	–	–	–
	No	85 (63.0)	**1.91 (1.15**–**3.17, p = 0.01)**	0.80 (0.54–1.17, p = 0.25)	1.13 (0.74–1.73, p = 0.57)

## Discussion

In this study, we found that patients that switched or continued their current systemic therapy after oligoprogression treated with metastasis-directed SBRT had comparable OS and PFS. These results could be indicative of the role of SBRT in combination with systemic therapy in the management of oligoprogression, suggesting that continuing systemic therapy beyond oligoprogression instead of changing to a further systemic treatment line may be feasible in selected patients and could reserve systemic treatment options for more fulminant disease progression.

The growing body of evidence on the use of SBRT in the treatment of oligoprogression has begun to shed light on the potential benefits and limitations of this approach across various malignancies. The randomized phase 2 CURB trial assessed the role of SBRT in oligoprogressive non-small-cell lung cancer (NSCLC) and breast cancer [11]. The majority of patients (75 % in standard-of-care arm and 85 % in SBRT arm) continued their systemic therapy after enrollment. The trial reported a significant PFS benefit in NSCLC patients receiving SBRT compared to the standard of care systemic therapy arm. However, no such PFS benefit was observed in the breast cancer cohort. Interestingly, the anatomical patterns of failure differed between the two primary tumor groups: Patients with breast cancer were more likely to develop new metastatic lesions, whereas most patients with NSCLC primarily progressed at pre-existing, non-irradiated lesions. Among NSCLC patients, the rate of failure at pre-existing lesions was reduced in the SBRT arm. Remarkably, liquid biopsy markers reflected these observations: while there was a significant decrease in blood-borne cell-free DNA content from baseline to follow-up in NSCLC patients receiving SBRT compared to those in the SBRT arm, no such differences between the two study arms were observed in breast cancer patients. The role of metastasis-directed SBRT for oligoprogressive breast cancer has consequently been questioned, also resonating with the negative randomized NRG-BR002 trial that did not show PFS or OS differences (currently only published in abstract form) [12]. On the other hand, the prospective single arm AVATAR trial (also only available in abstract form) found that SBRT could delay a switch of systemic therapy (combination of a CDK 4/6 inhibitor and an aromatase inhibitor therapy) in patients with oligoprogressive luminal breast cancer [13]. Further studies on oligoprogressive breast cancer patients are needed to assess which endpoints can be improved by metastasis-directed therapy, and to define which subgroups obtain the most benefit.

In a single-arm phase II trial focusing on metastatic renal cell carcinoma (mRCC), Hannan et al. reported promising results with SBRT extending the duration of ongoing systemic therapy by more than 6 months beyond disease progression in 70 % of patients [14]. This was echoed by Cheung et al., who investigated the impact of SBRT in the setting of oligoprogressive mRCC lesions in patients on tyrosine kinase inhibitor (TKI) therapy in a multicenter single-arm phase 2 trial [15]. The need to change systemic therapy was delayed for a median of over 1 year. The median PFS after SBRT was 9.3 months, with no grade 3–5 SBRT-related toxicities reported. While randomized data are awaited, these trials suggest SBRT may successfully delay further systemic treatment lines in patients with oligoprogressive mRCC.

A prospective, single-arm trial investigated the use of metastasis-directed therapy (SBRT or metastasectomy) in metastatic castration-resistant prostate cancer (mCRPC) patients with oligoprogression [16]. At a median follow-up of 6 months, 5 of the 20 enrolled patients were started on the next systemic therapy line, with a median next systemic therapy line-free survival of 12 months. For patients with oligoprogressive mCRPC, randomized trials are needed to confirm these encouraging findings.

While disease-specific trials on the role of metastasis-directed therapy in oligoprogressive patients are desirable, patient accrual is likely to be more challenging. On the other hand, a potential benefit of metastasis-directed therapy may be obscured in pan-cancer trials, despite enrolling patients easier. This is illustrated by the randomized phase 2 STOP trial, which assessed the impact of SBRT in patients with oligoprogression of any non-hematologic malignancy on PFS (published in abstract form) [17]. Initially designed to enroll only NSCLC patients, the trial was amended to be disease agnostic, given the poor accrual rates. In the currently available abstract, no difference in PFS and OS was observed between the SBRT and standard of care arm. These results may have been influenced by poor protocol adherence in the standard of care arm, where 10 % withdrew after randomization and an additional 23 % received SBRT.

The heterogeneity of patients with oligoprogression in general and included in our study warrants critical appraisal, as the potential treatment strategies and prognosis may differ: patients with intrinsic resistance - most consistent with the subgroup of patients with metachronous oligoprogression in our study - should likely be primarily managed by switching to an effective systemic agent, if possible using precision medicine and comprehensive molecular-pathological profiling of the disease. It is in particular patients with acquired resistance that may have a more favorable outcome and could be candidates of metastasis-directed therapy, as suggested in a large retrospective study including patients with metastatic lung cancer treated with anti-PD-(L)1 immunotherapy [18]. Acquired resistance presenting with oligoprogression is also a distinct pattern of failure among patients with oncogene-addicted NSCLC treated with TKIs [19], [20], [21], [22]. The ongoing randomized phase II HALT trial is investigating the benefit of adding metastasis-directed SBRT to continued TKI therapy in patients with oligoprogressive NSCLC and acquired TKI-resistance (NCT03256981).

While our study provides valuable insights into the outcomes and subsequent systemic treatment patterns in oligoprogressive patients who were treated with metastasis-directed SBRT, several limitations must be acknowledged. The subgroup of patients switching systemic therapy includes those who immediately start a new therapy and those who temporarily discontinue therapy, two disparate treatment strategies. These subgroups were deliberately combined to compare with the more experimental strategy of continuing therapy beyond disease progression. Most patients who discontinued or paused therapy eventually initiated subsequent treatment. The differing assumptions about disease progression that lead to these strategies warrant critical evaluation. For instance, continuing systemic therapy after ablation of progressive metastases suggests the presence of residual microscopic tumors hypothesized to be sensitive to the current therapy. Conversely, switching systemic therapy after SBRT implies an absence of further systemic benefit and the need for treatment intensification to prevent imminent progression, even after the visible tumor is ablated. Discontinuation of current systemic therapy without immediate initiation of the next line suggests that further widespread progression is not an immediate threat post-ablation of resistant lesions. Other reasons to discontinue may include side effects, patient preference, or limited availability of a promising next line, which might necessitate further molecular pathological examination after visible tumor ablation via SBRT.

Due to the retrospective nature of this study, it is subject to the inherent biases associated with such a design, including selection bias and possible confounding variables that were not identified or controlled for. The heterogeneity of the primary tumor entities, states of oligoprogression and therapy resistance included in the study may introduce confounding factors. Moreover, the group of patients switching systemic therapy combined those immediately starting their next line of systemic therapy, and those discontinuing or pausing systemic therapy. Due to the relatively large proportion of patients discontinuing systemic therapy, our data do not allow for a comparison of outcomes for upfront next line of systemic therapy compared with continuation beyond oligoprogression. The impact of starting a new systemic therapy upfront as opposed to delayed, at further progression following local metastases ablation, remains uncertain. A recent retrospective analysis of a prospective trial found that among oligorecurrent patients (i.e. not under systemic therapy at diagnosis of oligometastatic disease), delayed versus upfront start of systemic therapy resulted in shorter PFS yet without an impact on OS [23].

The precise cause for the cessation or interruption of systemic therapy in a specific patient cohort remains elusive. However, it is postulated that this phenomenon is more prevalent among patients with extensive prior treatments, or those exhibiting compromised fitness or organ function. Our analysis revealed a notable disparity in ECOG performance status scores between patients who immediately started a new systemic therapy after oligoprogression (9.1 % with ECOG performance status of 1–2) and those who discontinued or paused systemic therapy (35.7 % with ECOG performance status of 1–2). This interruption of systemic therapy, potentially necessitated by the patient's clinical condition, suggests that SBRT could serve as an interim therapeutic option, permitting the eventual resumption of systemic treatments under safer conditions.

Different types of cancer have different natural histories and responses to treatment, which may have affected the outcomes observed. It would be beneficial for future studies to focus on specific primary tumor entities to provide more precise information for clinical decision making in these particular groups.

## Conclusion

Oncological outcomes of patients that continued or switched systemic therapy after SBRT for oligoprogression were comparable, potentially indicating that further lines of treatment may be safely delayed in selected cases. Due to the heterogeneity of primary tumor types, states of oligoprogression, and therapy resistance, as well as the combined analysis of patients who either initiated a new line of systemic therapy or discontinued treatment, the observed outcomes should be interpreted with caution. Firm conclusions regarding the added benefit of SBRT in combination with systemic therapy, and the optimal strategy for systemic therapy continuation or alteration after SBRT in the setting of oligoprogression warrant assessment in perspective, randomized trials.

Conflict of Interest Statement for All Authors

Dr. Andratschke reports personal fees from AstraZeneca, personal fees from Debiopharm, grants, personal fees and non-financial support from ViewRay, grants and personal fees from Brainlab, outside the submitted work. Dr. Tanadini-Lang reports that her husband works at Varian Medical Systems. The other authors report no conflicts of interest.

## CRediT authorship contribution statement

**Jonas Willmann:** Conceptualization, Methodology, Formal analysis, Investigation, Data curation, Writing – original draft, Writing – review & editing, Visualization, Project administration. **Eugenia Vlaskou Badra:** Writing – review & editing, Investigation, Data curation. **Selma Adilovic:** Writing – review & editing, Investigation. **Maiwand Ahmadsei:** Writing – review & editing. **Sebastian M. Christ:** Writing – review & editing. **Stephanie Tanadini-Lang:** Writing – review & editing. **Michael Mayinger:** Writing – review & editing. **Matthias Guckenberger:** Resources, Writing – review & editing. **Nicolaus Andratschke:** Conceptualization, Methodology, Resources, Writing – original draft, Supervision, Project administration.

## Declaration of Competing Interest

The authors declare that they have no known competing financial interests or personal relationships that could have appeared to influence the work reported in this paper.
