# Supplementary material for: Stereotactic body radiotherapy for oligoprogression with or without switch of systemic therapy
Source: Clin Transl Radiat Oncol. 2024 Feb 23;45:100748. doi: 10.1016/j.ctro.2024.100748 (PMC10907512; doi:10.1016/j.ctro.2024.100748)

# Supplementary material

## Table A1

Radiotherapy treatment characteristics. Data are in n (%) or median (IQR). Abbreviations: GTV: gross tumor volume, cc: cubic centimeters.

|  |  | (n=135) |
| --- | --- | --- |
| **Fractions** | *Median (IQR)* | 5.0 (5.0 to 5.5) |
| **Dose per fraction (Gy)** | *Median (IQR)* | 7.0 (5.0 to 9.0) |
| **Total dose (Gy)** | *Median (IQR)* | 37.5 (35.0 to 40.2) |
| **Cumulative metastases volume (cc)** | *Median (IQR)* | 9.2 (3.6 to 28.1) |
| **Involved organ** | *Lung* | 43 (31.9) |
|  | *Bone* | 35 (25.9) |
|  | *Liver* | 25 (18.5) |
|  | *Lymph nodes* | 12 (8.9) |
|  | *Adrenal gland* | 11 (8.1) |
|  | *Pleura* | 5 (3.7) |
|  | *Soft tissue* | 3 (2.2) |
|  | *Spleen* | 1 (0.7) |

## Figure A1

Overall survival comparing patients that continue their previous systemic therapy, switch systemic therapy or discontinue/pause systemic therapy after SBRT. Band indicates 95% confidence interval. Abbreviations: STx, systemic therapy.


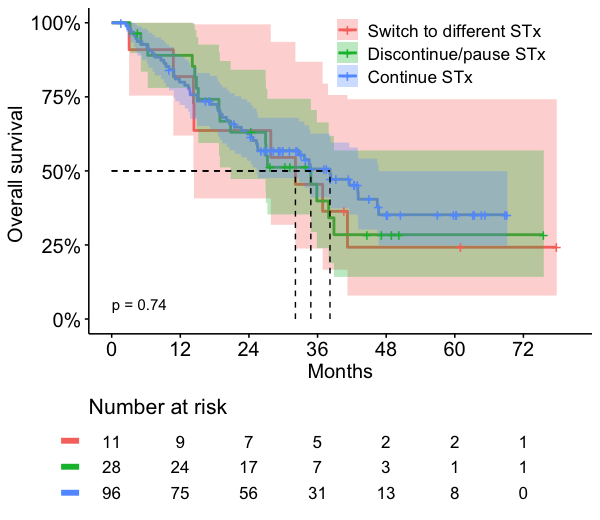

Supplement: Supplementary data 1 [file mmc1.docx]
