# Supplementary material for: Stereotactic body radiotherapy for oligoprogression with or without switch of systemic therapy
Source: Clin Transl Radiat Oncol. 2024 Feb 23;45:100748. doi: 10.1016/j.ctro.2024.100748 (PMC10907512; doi:10.1016/j.ctro.2024.100748)

Supplementary material

Table A1

Radiotherapy treatment characteristics. Data are in n (%) or median (IQR). Abbreviations: GTV: gross tumor volume, cc: cubic centimeters.

		(n=135)
**Fractions**	*Median (IQR)*	5.0 (5.0 to 5.5)
**Dose per fraction (Gy)**	*Median (IQR)*	7.0 (5.0 to 9.0)
**Total dose (Gy)**	*Median (IQR)*	37.5 (35.0 to 40.2)
**Cumulative metastases volume (cc)**	*Median (IQR)*	9.2 (3.6 to 28.1)
**Involved organ**	*Lung*	43 (31.9)
	Bone	35 (25.9)
	Liver	25 (18.5)
	Lymph nodes	12 (8.9)
	Adrenal gland	11 (8.1)
	Pleura	5 (3.7)
	Soft tissue	3 (2.2)
	Spleen	1 (0.7)

Figure A1

Overall survival comparing patients that continue their previous systemic therapy, switch systemic therapy or discontinue/pause systemic therapy after SBRT. Band indicates 95% confidence interval. Abbreviations: STx, systemic therapy.

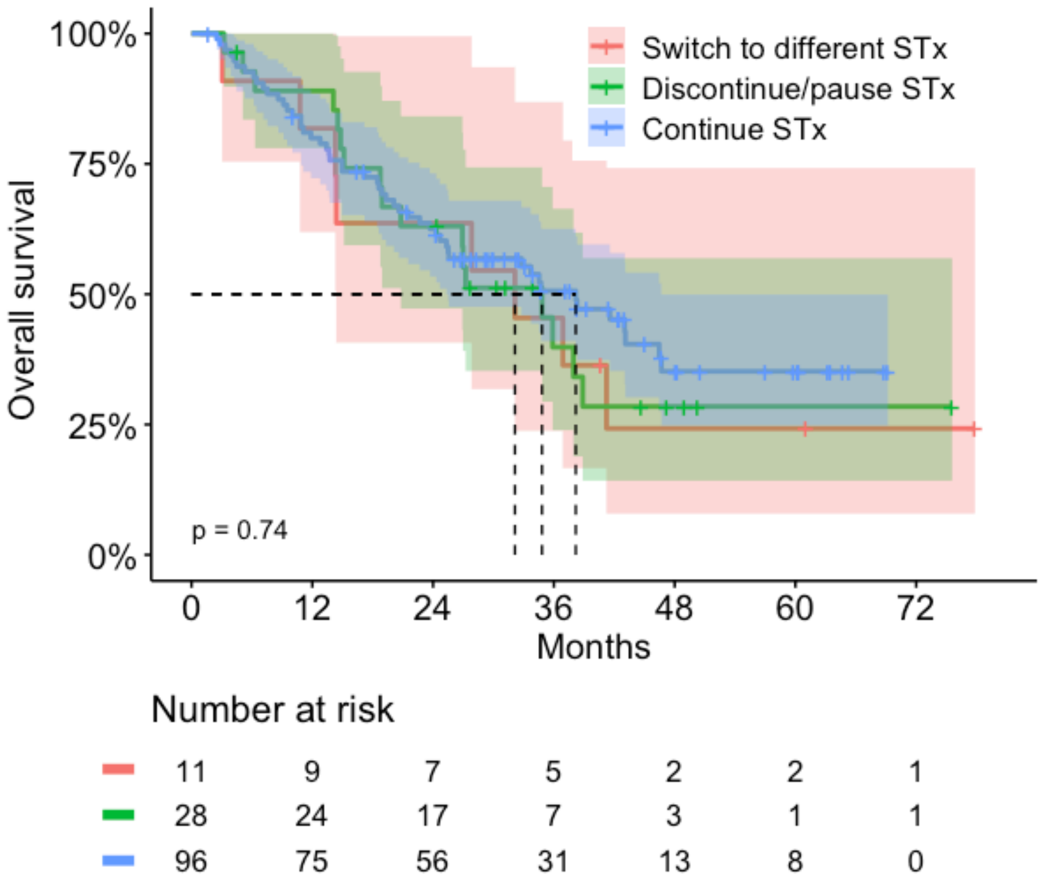

Supplement: Supplementary data 2 [file mmc2.pdf]
